# Venovenous extracorporeal life support for posttraumatic respiratory distress syndrome in adults: the risk of major hemorrhages

**DOI:** 10.1186/s13049-014-0056-0

**Published:** 2014-10-02

**Authors:** Meng-Yu Wu, Pyng-Jing Lin, Yuan-His Tseng, Kuo-Chin Kao, Hsuan-Ling Hsiao, Chung-Chi Huang

**Affiliations:** Department of Cardiovascular Surgery, Chang Gung Memorial Hospital and Chang Gung University, 5, Fushing Street, Gueishan Shiang, Taoyuan, 333 Taiwan; Department of Thoracic Medicine, Chang Gung Memorial Hospital and Chang Gung University, 5, Fushing Street, Gueishan Shiang, Taoyuan, 333 Taiwan; Department of Respiratory Therapy, Chang Gung Memorial Hospital and Chang Gung University, 5, Fushing Street, Gueishan Shiang, Taoyuan, 333 Taiwan; Department of Clinical Pharmacy, Chang Gung Memorial Hospital and Chang Gung University, 5, Fushing Street, Gueishan Shiang, Taoyuan, 333 Taiwan

**Keywords:** Extracorporeal life support, Extracorporeal membrane oxygenation, Posttraumatic acute respiratory distress syndrome, Blunt chest trauma, Heparin-minimized strategy

## Abstract

**Background:**

The aim of this retrospective study is to investigate the therapeutic benefits and the bleeding risks of venovenous extracorporeal life support (VV-ECLS) when used for adult posttraumatic respiratory distress syndrome (posttraumatic ARDS).

**Materials and methods:**

Twenty adult trauma patients (median age: 38 years, median injury severity score: 35) treated with VV-ECLS in a level I trauma center between January 2004 and June 2013 were enrolled in this study. The indication of VV-ECLS for posttraumatic ARDS was refractory hypoxemia (P_a_O_2_/F_i_O_2_ ratio ≤ 70 mmHg) under advanced mechanical ventilation. To minimize potential complications, a protocol-guided VV-ECLS was adopted.

**Results:**

Sixteen patients were weaned off VV-ECLS, and of these patients fourteen survived. Medians of the trauma-to-ECLS time, the pre-ECLS mechanical ventilation, and the ECLS duration in all patients were 64, 45, and 144 hours respectively. The median P_a_O_2_/F_i_O_2_ ratio was improved significantly soon after VV-ECLS, from 56 to 106 mmHg (*p* < 0.001). However, seven major hemorrhages occurred during VV-ECLS, of which three were lethal. The multivariate analysis revealed that the occurrence of major hemorrhages during VV-ECLS was independently related to the trauma-to-ECLS time < 24 hours (OR: 20; *p* = 0.02; 95% CI: 2–239; c-index: 0.81).

**Conclusions:**

Despite an effective respiratory support, VV-ECLS should be cautiously administered to patients who develop advanced ARDS soon after major trauma.

**Electronic supplementary material:**

The online version of this article (doi:10.1186/s13049-014-0056-0) contains supplementary material, which is available to authorized users.

## Introduction

Acute respiratory distress syndrome (ARDS) is a potentially lethal problem in trauma patients [1,2]. Based on the Berlin definition [3], the typical presentation of posttraumatic ARDS is a hypoxemic status [an arterial oxygen tension (P_a_O_2_)/fraction of inspired oxygen (FiO_2_) ratio ≤ 300 mmHg with a positive end-expiratory pressure (PEEP) ≥ 5 cmH_2_O] that is accompanied with bilateral pulmonary opacities, and occurs shortly after trauma. The common predisposing factors of posttraumatic ARDS are blunt thoracic injuries, traumatic shock requiring massive blood transfusion, and an injury severity score (ISS) ≥ 25 [2]. Similar to ARDS caused by nontraumatic etiologies, posttraumatic ARDS is primarily treated with mechanical ventilation. To reduce the injurious effects of cyclical inflation and deflation on the already injured lungs during positive pressure ventilation, lung-protective ventilation is preferred among ARDS patients, using low tidal-volumes (≤6 mL/kg/min) and optimal PEEPs to achieve an inspiratory plateau pressure (P_plt_) ≤ 30 cmH_2_O [4,5]. However increases in FiO_2,_ PEEP, and P_plt_ may be unavoidable when an acceptable arterial oxygenation cannot be maintained. The hyperinflated-hyperoxic ventilation may exacerbate pulmonary shunting and induce a repeated mechanical-biological trauma [6]. This ventilator-induced lung injury may initiate a vicious cycle that leads to severe ARDS (P_a_O_2_/FiO_2_ ratio ≤ 100 mmHg) with multiple organ dysfunctions [7]. Venovenous extracorporeal life support (VV-ECLS) may break this vicious cycle by conducting a prepulmonary blood gas exchange to share the workload with native lung, which enables physicians to continue lung-protective ventilation [8]. Nevertheless, because of its inherent thrombogenicity owing to the blood-surface interaction [9], VV-ECLS requires systemic heparinization and involves a 40% risk of hemorrhage at intracranial, surgical, and cannulation sites [10]. This risk may increase when VV-ECLS is administrated to patients who have just sustained major trauma and damage-control interventions. These patients tend to have the trauma-induced coagulopathy (TIC) [11] and may be vulnerable to heparinization. To evaluate the therapeutic benefits and bleeding risks of VV-ECLS when used for posttraumatic ARDS, nine years of experience with this therapy was reviewed.

## Materials and methods

### Study population

From January 2004 to June 2013, a total of 561 patients received ECLS for hemodynamic (venoarterial mode; n = 437) or pulmonary (VV mode; n = 124) support at Chang Gung Memorial Hospital. Among the 124 patients, 20 adults had posttraumatic ARDS and were enrolled in this retrospective study. The institutional review board of the hospital approved the protocol (CGMF IRB no. 102-4437B) and waived the necessity of individual patient consent.

### Therapeutic protocol: conventional mechanical ventilation

The definition of posttraumatic ARDS was based on the Berlin definition [3]. Conventional mechanical ventilation was used as the primary respiratory support for posttraumatic ARDS and a lung-protective strategy was adopted under paralytic sedation. The protocol of conventional mechanical ventilation for posttraumatic ARDS was demonstrated in our previous report [12] and showed in Additional file 1. When conventional mechanical ventilation alone was thought insufficient for an adequate arterial oxygenation (often a P_a_O_2_/FiO_2_ ratio < 70 mmHg with a FiO_2_ ≥ 0.8), VV-ECLS was administered. The exclusion criteria of VV-ECLS were (1) an uncontrolled hemorrhagic shock; (2) an identified acute heart failure; or (3) a severe traumatic brain injury. A whole body computed tomography was suggested to all candidates to exclude undetected internal hemorrhages before the administration of VV-ECLS.

### Implantation of VV-ECLS

As reported previously [13], we used the Capiox emergent bypass system (Terumo Inc., Tokyo, Japan) with a heparin-coated inner surface as our ECLS device. Since a double lumen catheter was not available during the period of study, two wire-wound polyurethane vascular cannulae (DLP Medtronic, Minneapolis, MN, USA; inflow: 19–23 French, outflow: 17–21 French) were used to established the femoral (in)-jugular (out) VV-ECLS via percutaneous cannulation. The ECLS gas flow rate was initially set high (10 L/min, pure oxygen), and the blood pump speed was gradually increased to achieve a pulse oximetry-detected oxyhemoglobin saturation (S_p_O_2_) ≥ 90%. If necessary, a modest volume replacement (preferably packed red blood cell) was used to improve an unsteady ECLS blood flow. The positions of the cannula tips were confirmed with a chest-abdominal radiograph in order to prevent a substantial recirculation.

### Therapeutic Protocol of VV-ECLS in posttraumatic ARDS

**Figure 1:**
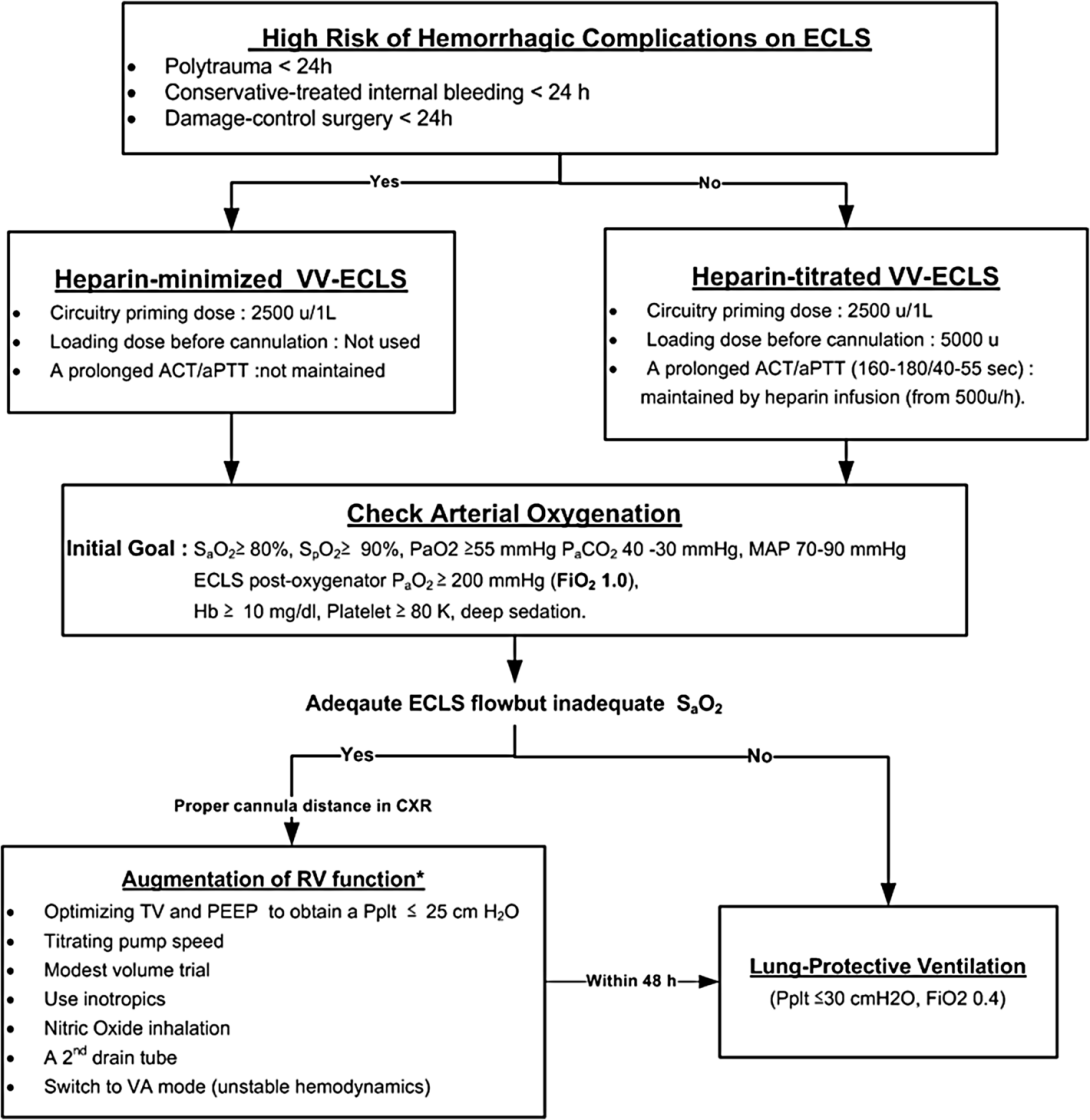
**The therapeutic protocol of venovenous extracorporeal life support in patients with posttraumatic acute respiratory distress syndrome.** ACT: Active clotting time. aPTT: Activated partial thromboplastin time. PEEP: Positive end-expiratory pressure. S_p_O_2_: Pulse oximetry-detected oxyhemoglobin saturation. P_a_O_2_: Arterial oxygen tension. P_a_CO_2_: Arterial carbon dioxide tension. MAP: Mean arterial pressure. Hb: Hemoglobin. FiO_2_: The fraction of inspired oxygen. TV3: Tidal volume. P_plt_: Inspiratory plateau pressure.

Figure 1 illustrates our VV-ECLS protocol for posttraumatic ARDS. Anticoagulation, oxygenation, and ventilation were three highlighted points. Before implantation, the ECLS specialists must evaluate the risks and benefits of administering a fully heparinized (heparin-titrated) VV-ECLS to the trauma patient. Based on our experience of postcardiotomy ECLS [14], we routinely administer the heparin-minimized ECLS to patients who require ECLS within 24 hours after major surgeries or trauma [15], since these patients are at high risk for consumption coagulopathies. TIC is a consumption coagulopathy specific to trauma patients, and is defined as a hypocoagulable state [prothrombin time (PT)- international normalized ratio (INR) ≥ 1.2 or the activated partial thromboplastin time (aPTT) ≥ 35 seconds] presented soon after trauma [16,17]. The active clotting time (ACT) and aPTT were checked as needed, at least every 6 hour in the first day of VV-ECLS. In heparin-titrated ECLS, the therapeutic range of the ACT/aPTT value was 160–180/40–55 seconds, and heparin was administered intravenously when the aPTT value dropped below 40 seconds. Patients on the heparin-minimized ECLS received no exogenous heparinization. The heparin-minimized ECLS maintained a high blood flow rate (>3 L/min) and was switched to the heparin-titrated mode after 48 hours to reduce the possibility of thromboembolism. To achieve acceptable oxygenation and to reduce the bleeding risk, hemaglobin and platelet count were also checked daily to maintain a level more than 10 g/dL and 80 billion/L, respectively. Coagulation factor transfusion was seldom needed in patients without hemorrhage. In patients with hemorrhages, withholding heparin plus blood transfusion (RBC: plasma: platelet about 1:1:3) was the first step to achieve hemostasis on ECLS. Endoscopic, angiographic or surgical hemostasis was launched with low threshold once the conservative treatment failed or was impossible to stablize the hemorrhage. When an obvious anisocoria (a diameter difference > 2 mm) was detected, a brain computed tomography was immediately performed to assess the possibility of ICHs. VV-ECLS was withdrawn (or rapidly weaned) after obtaining the family’s permission when a major ICH was identified. The choice whether to perform a surgical decompression depended on the opinion of the consulting neurosurgeon.

The adequacy of oxygenation during VV-ECLS was checked by repeated arterial blood gas samplings, at least every 6 hours during the first day. With an improvement of S_p_O_2_, the lung-protective ventilation was continued with a decreased FiO_2_, respiratory rate, and P_plt_. However attempts to step-down the FiO_2_ may be hindered by an unsatisfactory S_p_O_2_ level in spite of a well-functioning VV-ECLS. A substantial recirculation of the outflow (oxygenated) blood of VV-ECLS was often found in this scenario and needed to be corrected quickly [18]. Figure 1 also outlines common methods of reducing the recirculation during VV-ECLS. After optimizing the oxygenation, it was critical to remove the excessive lung water with diuretics or continuous venovenous hemofiltration in order to improve the pulmonary compliance [19]. Weaning from the ECLS was tried once significant pulmonary improvements were shown. The pump flow was tapered to 2.0 L/min then the FiO_2_ of the ECLS gas flow was turned to 0.21. Decannulation was performed after 4 to 6 hours if the patient remained stable. The weaning process would be stopped if (1) hemodynamic instability, or (2) a P_a_O_2_/FiO_2_ ratio < 100 mmHg or P_a_CO_2_ ≥ 60 mmHg under lung protective ventilation with a maximal FiO_2_ of 0.6.

### Statistical analysis

Statistical analyses were performed using SPSS for Windows (Version 15.0, SPSS, Inc., IL, USA). Because the dataset was small, nonparametric methods including the Mann–Whitney *U* or Wilcoxon signed ranks tests was used to conduct univariate comparisons of the independent or paired continuous variables. The Chi-square or Fisher’s exact test was used to compare the categorical variables. The level of statistical significance was set at *p* < 0 .05. Continuous variables with a *p* < 0.05 were dichotomized on the basis of cut-off values [14]. The cut-off values were determined by the receiver operating characteristic curve (ROC) analysis. These dichotomized risk factors were tested by the multivariate logistic regression analysis to identify independent predictors of in-hospital mortality and occurrence of major hemorrhages.

## Results

### Demographics

Table 1 summarizes the trauma characteristics, pre-ECLS managements, and outcomes of the twenty patients. A combination of high-grade thoracoabdominal injuries were common and resulted in a high median ISS (35; IQR: 21–50). The median durations of the pre-ECLS mechanical ventilation and the trauma-to-ECLS time were 45 (IQR: 8–148) hours and 64 (IQR: 12–230) hours, respectively. Fifteen patients received damage-control interventions, including thoracotomy (including subxiphoid incision; n = 7), laparotomy (n = 7), or transcatheter arterial embolization (n = 7) before VV-ECLS. Four patients were diagnosed with minor intracranial hemorrhages (ICH in Cases 4 and 14, SAH in Cases 12 and 15) before VV-ECLS. Sixteen patients were weaned off VV-ECLS and fourteen survived. The six nonsurvivors died of sepsis (n = 3) or hemorrhagic complications during ECLS (n = 3). Table 2 lists the results of univariate comparisons of clinical parameters between different patient groups. Despite there being no independent risk factor of in-hospital mortality identified, a trauma-to-ECLS time ≤ 15 hours [Sensitivity (Sn): 71%; Specificity (Sp): 92%; Positive Predictive Value (PPV): 83%; Negative Predictive Value (NPV): 86%; area under ROC curve (AUROC): 0.82; *p* = 0.02] was found to be the independent predictor of hemorrhage during VV-ECLS [odd ratio (OR): 30, *p* = 0.01, 95% confidence intervals (CI): 2–441]. For practical purposes, we also tested the predictive power of a trauma-to-ECLS time ≤ 24 hours. Acceptable predictive power was also obtained in this cut-off point (Sn: 86%; Sp: 77%; PPV: 67%, NPV: 91%, AUROC: 0.81; *p* = 0.02). The OR, *p* value, and 95% CI at this point were 20, 0.02, and 2–239, respectively.

**Table 1:** **Summary of the characteristics of trauma, types of damage-control interventions, and outcomes**

**No**	**Age (year)**	**Mechanism of trauma**	**ISS**	**Pre-ECLS management**		**Trauma to ECLS (hour)**	**Outcome (ECLS hour)**
				**MV hour**	**Damage control surgery/TAE**		
1	53	Car-pedestriant accident	20	4	No	5	Survived (69)
2	29	Motorbike accident	17	6	No	7	Withdrawn-and-survived (61)
3	33	Motorbike accident	29	5	**Thoracotomy**: RML-RLL bilobectomy, pericardiotomy	9	Survived (187)
4	38	Motorbike accident	50	8	No	10	Weaned-and-survived (145)
5	59	Car accident	75	9	Pericardiocentesis and **subxiphoid incision** to release cardiac tamponade	11	Survived (75)
6	23	Accidental fall	75	9	**Thoracotomy**: Repair RML/RLL lacerations	14	Withdrawn-and-dead (40)
					**Laparotomy**: Ligation of right hepatic artery with perihepatic packing		
7	49	Trunk-pedestriant accident	41	14	**TAE** for retroperitoneal hemorrhage, external fixation of pelvic fracture	16	Survived (222)
8	43	Motorbike accident	29	4	No	21	Survived (77)
9	20	Motorbike accident	75	21	**TAE** for grade 4 hepatic laceration.	23	Dead-on-ECLS (24)
					**Laparotomy**: Ligation of right hepatic artery and right portal vein with perihepatic packing		
10	57	Car accident	75	49	**TAE** for retroperitoneal hemorrhage, external fixation of pelvic fracture.	52	Dead-on-ECLS (423)
					**Laparotomy**: Resect terminal ileum and right colon with end-ileostomy for bowel perforation		
11	37	Accidental fall with chest penetrating injury	22	74	**Laparotomy**: Repair gastric and diaphragmatic perforation, repair spleen avulsion	76	Survived (66)
12	61	Motorbike accident	43	91	**Laparotomy**: Repair small bowel perforation	100	Survived (143)
13	32	Motorbike accident	29	126	Internal fixation of right proximal femoral fracture	138	Dead-on-ECLS (1030)
14	28	Car accident	43	143	**TAE** for grade 3 hepatic laceration	175	Survived (94)
					**Laparotomy**: Repair mesocolonic laceration		
15	72	Motorbike accident	18	227	**Thoracoscopy** for empyema evacuation	248	Dead-on-ECLS (111)
16	56	Motorbike accident	10	150	No	295	Survived (517)
17	25	Trunk-pedestriant accident	41	365	**TAE** for grade 3 hepatic laceration	384	Survived (456)
					**Thoracotomy**: RML -RLL bilobectomy and bronchoplasty		
					**Exploratory laparotomy**		
18	27	Motorbike accident	50	174	**TAE** for grade 5 hepatic laceration	574	Weaned-but-Dead (352)
					**Laparoscopy** for persistent bile leak and intra-abdominal abscess		
					**Thoracotomy**: Descending aortic replacement		
19	27	Compression injury	25	40	**Laparotomy**: Cystorrhaphy	115	Survived (161)
20	58	Accidental fall	17	310	**TAE** for grade 4 renal laceration	334	Survived (169)

**ECLS**: Extracorporeal life support.

**MV hour**: Mechanical ventilation hour before and extended to ECLS without interruption. **TAE**: Transcatheter arterial embolization.

**CA**: Cardiac arrest. **RML**: Right middle lobe of lung, **RLL**: Right lower lobe of lung.

**Table 2:** **Demographic and clinical data**

**Variable**	**Survivors (n = 14)**	**Non-survivors (n = 6)**	***p***	**Patient with hemorrhagic complication (n = 7)**	**Patient without hemorrhage complications (n = 13)**	***p***
**Age (years)**	41 (29–57)	30 (22–61)	0.35	33 (23–53)	43 (28–58)	0.49
**Injury severity score**	29 (19–43)	63 (26–75)	0.09	29 (18–75)	41 (24–47)	1.0
**Pre-ECLS CPR (n)**	1	3	0.06	2	2	0.59
**Pre-ECLS ICH (n)**	2	2	0.55	2	2	0.59
**Trauma-to-ECLS hour**	49 (10–205)	95 (21–329)	0.35	10 (7–23)	115 (37–315)	0.008*
**Trauma-to-ECLS < 24 hours (n)**	2	7	0.64	6	3	0.02*
**Pre-ECLS intubation hour**	27 (6–145)	88 (18–187)	0.31	8 (5–21)	91 (27–162)	0.04*
**Pre-ECLS RBC transfusion** ^†^ ** (u)**	1 (0–4)	10 (1–18)	0.34	6 (3–17)	1 (0–3)	0.05*
**Pre-ECLS platelet transfusion (u)**	0	6 (0–39)	0.1	0 (0–18)	0	0.58
**Pre-ECLS plasma transfusion** ^**‡**^ ** (u)**	5 (3–16)	11 (0–17)	0.18	5 (3–16)	2 (0–6)	0.05*
**Pre-ECLS TIC**	9	6	0.26	6	9	0.61
**Heparin-minimized ECLS (n)**	7	4	0.64	7	4	0.005*
**ECLS pump flow (L/min)**	4 (3.6-4.6)	3.7 (3.4-4)	0.27	4.1 (3.3-4.3)	3.6 (3.5-4.6)	0.7
**1** ^**st**^ **day RBC transfusion** ^†^ ** (u)**	1 (1–10)	7 (3–13)	0.16	10 (3–14)	1 (1–6)	0.007*
**1** ^**st**^ **day Platelet transfusion (u)**	0 (0–24)	12 (12–24)	1.0	24 (12–24)	0 (0–12)	0.03*
**1** ^**st**^ **day Plasma transfusion** ^**‡**^ ** (u)**	11 (5–22)	10 (0–24)	0.26	11 (5–22)	0 (0–7)	0.05*
**CVVH on ECLS (n)**	3	4	0.12	1	6	0.33
**Hemorrhagic complications (n)**	4	3	0.61	-	-	-
**Weaning-off ECLS (n)**	14	2	0.003*	5	11	0.59
**ECLS hour**	144 (74–196)	232 (36–575)	0.9	69 (40–145)	169 (86–440)	0.02*
**Post-ECLS intubation hour**	231 (61–476)	-	-	72 (0–363)	111 (23–427)	0.7
**Tracheostomy (n)**	7	1	0.33	2	6	0.64
**Hospital days**	69 (27–81)	32 (4–46)	0.04*	45 (5–76)	45 (27–78)	0.49

Numerical variable is presented as median and interquartile range (IQR).

**ECLS**: Extracorporeal life support. **CPR**: Cardiopulmonary resuscitation. **ICH**: Intracranial hemorrhage. **Trauma-to-ECLS hour**: The time interval between trauma to the initiation of ECLS. ^†^RBC transfusion includes the amount of whole blood and packed red cell concentrate. ^**‡**^Plasma transfusion includes the amount of whole blood, frozen fresh plasma and cryoprecipitate. **TIC**: Trauma induced coagulopathy (INR > 1.3 or aPTT > 35 seconds). **CVVH**: Continuous venovenous hemofiltration. *p < 0.05.

### Improvements of arterial gas exchange

A series of changes in P_a_O_2_/FiO_2_ ratio during VV-ECLS between different patient groups was demonstrated in Table 3. An improved oxygenation was showed in all patients soon after the administration of VV-ECLS. After testing by the Wilcoxon signed ranks test, the increase of P_a_O_2_/FiO_2_ ratio was significant in survivors (*p* = 0.004), non-survivors (*p* = 0.03), patients with hemorrhages (*p* = 0.003), and patients without hemorrhages (*p* = 0.005). However, only the survivors showed a continuous increase in P_a_O_2_/FiO_2_ ratio in the first day of VV-ECLS (*p* = 0.01).

**Table 3:** **Laboratory data associated with gas exchange and coagulation**

**Variable**	**Survivors (n = 14)**	**Non-survivors (n = 6)**	***p***	**Patient with hemorrhagic complication (n = 7)**	**Patient without hemorrhage complications (n = 13)**	***p***
**Pre-ECLS data**						
PaO_2_ (mmHg)	56 (48–73)	55 (42–67)	0.55	53 (42–66)	58 (49–69)	0.59
PaO_2_/FiO_2_ ratio (mmHg)	56 (48–73)	57 (42–68)	0.66	53 (42–66)	58 (49–70)	0.7
PaCO_2_ (mmHg)	61 (47–89)	52 (41–76)	0.64	49 (40–78)	52 (49–91)	1.0
Hemoglobin (g/dL)	11 (10–13)	9 (8–11)	0.06	10 (9–15)	11 (9–12)	0.64
Platelet count (×10^9^/L)	164 (90–193)	88 (51–34)	0.11	172 (107–183)	115 (67–193)	0.76
INR of Prothrombin Time > 1.2	7	6	0.05*	5	8	1.0
aPTT > 35 (seconds)	7	3	1.0	5	5	0.35
**Data collected on ECLS at1 hour**						
PaO_2_/FiO_2_ ratio (mmHg)	108 (65–103)	103 (71–151)	0.84	99 (166–146)	110 (74–171)	0.54
PaCO_2_ (mmHg)	37 (30–46)	39 (31–45)	0.46	42 (35–44)	35 (27–48)	1.0
Hemoglobin g/dL)	10 (9–11)	10 (9–14)	0.72	10 (8–12)	10 (9–11)	0.76
Platelet count (×10^9^/L)	98 (65–153)	100 (45–134)	0.72	99 (31–142)	100 (64–160)	0.54
INR of Prothrombin Time > 12^†^	2	1	1.0	3	0	0.03*
aPTT > 120^†^ (seconds)	10	6	0.27	6	10	1.0
**at24 hour**						
PaO_2_/FiO_2_ ratio (mmHg)	190 (100–246)	93 (86–262)	0.44	98 (86–238)	180 (96–252)	0.59
Hemoglobin (g/dL)	10 (9–11)	10 (9–12)	0.6	10 (8.8-12)	10 (9–11)	0.82
Platelet count (×10^9^/L)	99 (63–139)	76 (62–92)	0.18	75 (62–93)	96 (73–131)	0.18
**Before ECLS weaning**						
PaO_2_/FiO_2_ ratio (mmHg)	261 (186–338)	-	-	229 (135–256)	288 (178–343)	0.18

Numerical variable is presented as median and interquartile range (IQR).

**ECLS**: Extracorporeal life support. **PaO**_**2**_ **/FiO**_**2**_ **ratio**: The arterial oxygen tension **(PaO**_**2**_ **)** divided by the fraction of inspired oxygen **(FiO**_**2**_ **)**. **INR**: International normalized ratio. **aPTT**: Activated partial thromboplastin time. **TIC**: Trauma induced coagulopathy (INR > 1.3 or aPTT > > 35). *p < 0.05. ^†^The maximum of the detectable values.

### The Incidence of Hemorrhaging during ECLS

**Figure 2:**
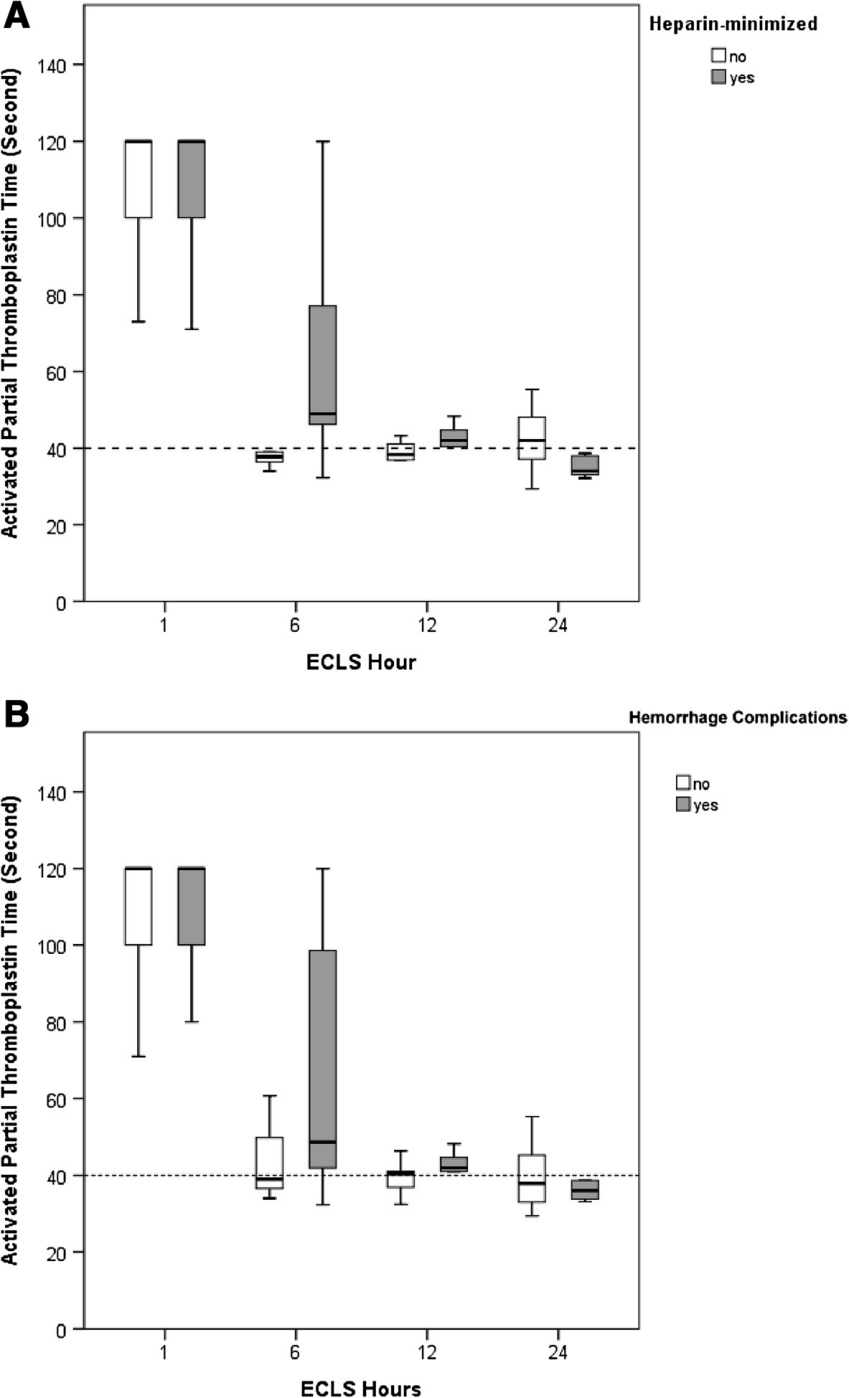
**The time courses of the activated partial thromboplastin time during the first 24 hours of venovenous extracorporeal life support.** Median with 25^th^ to 75^th^ interquatile range. *The upper limit of detectable value of aPTT is 120 seconds.

Eleven patients (Cases 1–10 and Case 15 for just receiving surgery for empyema) fulfilled the criteria and received heparin-minimized VV-ECLS for 48 hours. Seven patients (six with TIC) had major hemorrhages during VV-ECLS, after a median ECLS duration of 12 (4–61) hours. All of the seven patients received heparin-minimized VV-ECLS as a prophylactic measure. The clinical presentations and management of the seven hemorrhagic complications are summarized in Table 4. A series of changes in coagulation profiles during VV-ECLS between different patient groups was also demonstrated in Table 3. To investigate the response of the patients’ coagulation system to different ECLS strategies, aPTT data collected during the first day of VV-ECLS are presented as box plots in Figure 2.

**Table 4:** **Management of Hemorrhagic Complications on venovenous extracorporeal life support**

**Case**	**Clinical presentations (Hours after ECLS initiation)**	**Hemorrhagic site**	**Intervention on ECLS (Outcome)**
**1**	Hemorrhaging from the open fracture wound of sternum (2)	Fractured sternum	Sternotomy (Survived)
**2**	Anisocoria (61)	Multiple intracranial hemorrhages	Craniotomy (Survived)
**3**	Abdominal compartment syndrome (IAP * > 30 cm H_2_O) (12)	The conservatively-treated liver laceration	Peritoneal drainage (Survived)
**4**	Anisocoria (71)	Intraventricular hemorrhage	No (Survived)
**6**	Hemorrhaging from the decompressive laparotomy (12)	The packed liver laceration	Laparotomy (Dead)
	Anisocoria (40)	Multiple Intracranial hemorrhages.	
**9**	Hemorrhaging from the decompressive laparotomy (4)	The packed liver laceration	No (Dead)
**15**	Hemorrhaging from the chest tube (15)	The decorticated lung	Thoracotomy (Dead)

*IAP: Intra-abdominal pressure.

## Discussion

This study aimed to investigate the therapeutic benefits and the safety of VV-ECLS when used for adult posttraumatic ARDS, since the ECLS-associated coagulopathy may induce severe consequences in trauma patients. As seen in previous reports [20,21] and the current study, VV-ECLS is an efficient device to improve the blood gas exchange and may achieve a survival rate of more than 70% in adult patients with severe posttraumatic ARDS. The incidence of major hemorrhages during VV-ECLS is quite low in patients receiving VV-ECLS after several days of trauma, as seen in patients supported for non-traumatic ARDS [10]. According to a recent study reported by Ried et al. [20], the incidence of major hemorrhages during extracorporeal lung support (n = 52; 26 used VV-ECLS) is only 4% (n = 2) in patients with a median trauma-to-ECLS time of 4.5 days. However, the therapeutic challenge here is administering VV-ECLS to patients at high risk for TIC. Despite being guided by a comprehensive protocol, the current study still yielded an incidence of major hemorrhages of 35% (n = 7) in 20 patients with a median trauma-to-ECLS time of 64 hours (2.7 days). The median trauma-to-ECLS time was only 10 hours in the group of hemorrhage. To explain the high incidence of major hemorrhages, we analyzed the changes of blood coagulability during VV-ECLS in order to evaluate the interaction between TIC and ECLS-associated coagulopathy. Since there is little literature information about this issue, the current report should provide valuable information to ECLS specialists who attempt to enroll patients that have just sustained major trauma or damage-control interventions.

As shown in Figure 2, all patients experienced an extremely hypocoagulable state soon after being connected to an ECLS circuit. The suppression of blood coagulability decayed with time and almost disappeared after 12 hours in most of the patients. This phenomenon seemed to be compatible with the pharmacodynamics of heparin. Except the initial hypocoagulability, all patients showed a decreased platelet count after 24 hours of ECLS. This platelet consumption was also found in previous studies and could be explained by an increased volume of distribution and blood-surface interaction during extracorporeal circulation [9,13]. Therefore we might suggest that the ECLS-associated coagulopathy was a profound hypocoagulable state induced by heparinization, hemodulition and platelet consumption [9]. This heparin-induced hypocoagulability may persist for 12 hours in adult patients with a relatively normal coagulability before VV-ECLS. From this viewpoint, reducing or even pharmacologically neutralizing the heparin content at the beginning of VV-ECLS should be helpful to attenuate the anticoagulant impact of ECLS.

In regards to the interaction between TIC and ECLS-associated coagulopathy, we had some indirect findings. As we were limited by the small number of cases, we failed to identify the correlation between TIC and the major hemorrhage during VV-ECLS, but we did find that the blood coagulability (both the intrinsic and external coagulation pathways) and the platelet counts were more suppressed in the hemorrhage group than in the non-hemorrhage group at the beginning of ECLS. Patients in the hemorrhage group also had more blood transfusions in the day before ECLS. Therefore, we may assume that these patients have a more severe TIC and are very sensitive to ECLS-associated coagulopathy, even to the heparin-minimized ECLS. The two coagulopathies may exert a synergistic effect strongly against blood coagulation and cause diffuse hemorrhages from the crushed solid organs and soft tissues. Despite there might be room for hemostasis with transfusion therapy alone, a proactive stance of surgical interventions should be taken toward major hemorrhages during ECLS. Not only because hemorrhages may reduce the efficacy of gas exchange on VV-ECLS by decreasing the pump flow rate and the amount of hemoglobin [22], but also as the transfusion therapy itself may also worsen the existed pulmonary injury.

The limitations of this study are its retrospective design and the small number of cases involved. Further prospective and collaborative studies involving large populations and an integrated protocol of coagulation measurements (such as the thrombelastogram) are necessary to optimize the analysis of the therapeutic effects of VV-ECLS.

## Conclusion

VV-ECLS is an effective respiratory support in adult posttraumatic ARDS. However, the risk of major hemorrhage during VV-ECLS may be significant in patients who have just sustained major trauma and have a packed or conservatively treated hemorrhage.

## Additional file

Additional file 1:
**The integrated protocol of conventional mechanical ventilation and venovenous extracorporeal life support for posttraumatic ARDS.** The part of conventional ventilation was modified from the method described in the reference [12].
