# Supplementary material for: Venovenous extracorporeal life support for posttraumatic respiratory distress syndrome in adults: the risk of major hemorrhages
Source: Scand J Trauma Resusc Emerg Med. 2014 Oct 2;22:56. doi: 10.1186/s13049-014-0056-0 (PMC4189614; doi:10.1186/s13049-014-0056-0)
Supplement: Additional file 1: — The integrated protocol of conventional mechanical ventilation and venovenous extracorporeal life support for posttraumatic ARDS. The part of conventional ventilation was modified from the method described in the reference [12]. [file 13049_2014_56_MOESM1_ESM.pdf]

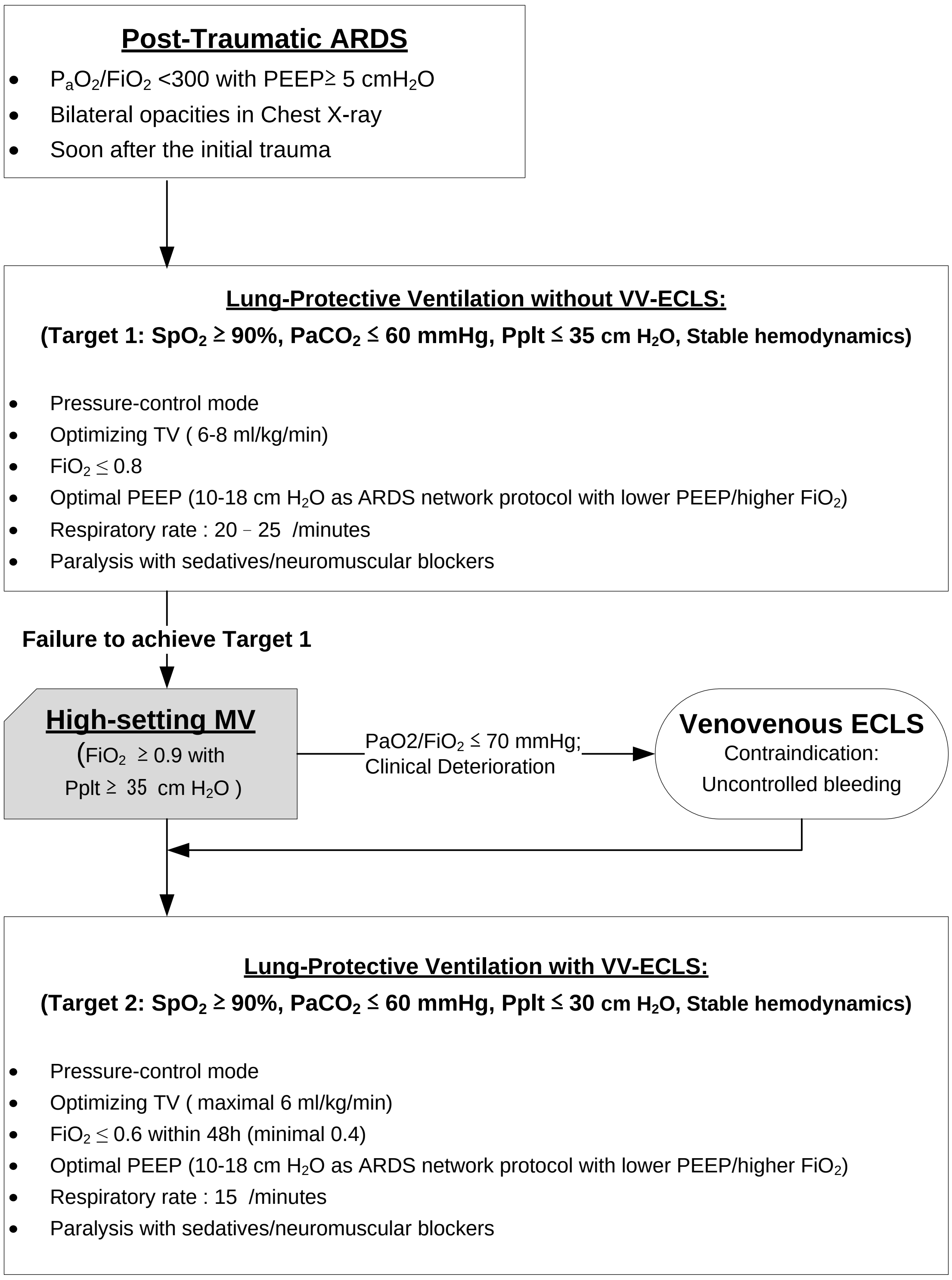
Post-Traumatic ARDS
P_aO_2/FiO_2 <300 with PEEP≥ 5 cmH_2O
Bilateral opacities in Chest X-ray
Soon after the initial trauma
Lung-Protective Ventilation without VV-ECLS:
(Target 1: SpO_2 ≥ 90%, $PaCO_2$ ≤ 60 mmHg, Pplt ≤ 35 cm H_2O, Stable hemodynamics)
Pressure-control mode
Optimizing TV (6-8 ml/kg/min)
FiO_2 ≤ 0.8
Optimal PEEP (10-18 cm H_2O as ARDS network protocol with lower PEEP/higher FiO_2)
Respiratory rate : 20 – 25 /minutes
Paralysis with sedatives/neuromuscular blockers
Failure to achieve Target 1
High-setting MV
(FiO_2 ≥ 0.9 with
Pplt ≥ 35 cm H_2O)
PaO2/FiO_2 ≤ 70 mmHg;
Clinical Deterioration
Venovenous ECLS
Contraindication:
Uncontrolled bleeding
Lung-Protective Ventilation with VV-ECLS:
(Target 2: SpO_2 ≥ 90%, $PaCO_2$ ≤ 60 mmHg, Pplt ≤ 30 cm H_2O, Stable hemodynamics)
Pressure-control mode
Optimizing TV (maximal 6 ml/kg/min)
FiO_2 ≤ 0.6 within 48h (minimal 0.4)
Optimal PEEP (10-18 cm H_2O as ARDS network protocol with lower PEEP/higher FiO_2)
Respiratory rate : 15 /minutes
Paralysis with sedatives/neuromuscular blockers
